# Ginseng pharmacology: a new paradigm based on gintonin-lysophosphatidic acid receptor interactions

**DOI:** 10.3389/fphar.2015.00245

**Published:** 2015-10-27

**Authors:** Sun-Hye Choi, Seok-Won Jung, Byung-Hwan Lee, Hyeon-Joong Kim, Sung-Hee Hwang, Ho-Kyoung Kim, Seung-Yeol Nah

**Affiliations:** ^1^Ginsentology Research Laboratory, Department of Physiology, College of Veterinary Medicine, Konkuk University, Seoul, South Korea; ^2^Department of Pharmaceutical Engineering, Sangji University, Wonju, South Korea; ^3^Mibyeong Research Center, Korea Institute of Oriental Medicine, Daejeon, South Korea

**Keywords:** ginseng, gintonin, LPAs, G protein-coupled LPA receptors, ginseng pharmacology, new paradigm

## Abstract

Ginseng, the root of *Panax ginseng,* is used as a traditional medicine. Despite the long history of the use of ginseng, there is no specific scientific or clinical rationale for ginseng pharmacology besides its application as a general tonic. The ambiguous description of ginseng pharmacology might be due to the absence of a predominant active ingredient that represents ginseng pharmacology. Recent studies show that ginseng abundantly contains lysophosphatidic acids (LPAs), which are phospholipid-derived growth factor with diverse biological functions including those claimed to be exhibited by ginseng. LPAs in ginseng form a complex with ginseng proteins, which can bind and deliver LPA to its cognate receptors with a high affinity. As a first messenger, gintonin produces second messenger Ca^2+^ via G protein-coupled LPA receptors. Ca^2+^ is an intracellular mediator of gintonin and initiates a cascade of amplifications for further intercellular communications by activation of Ca^2+^-dependent kinases, receptors, gliotransmitter, and neurotransmitter release. Ginsenosides, which have been regarded as primary ingredients of ginseng, cannot elicit intracellular [Ca^2+^]_i_ transients, since they lack specific cell surface receptor. However, ginsenosides exhibit non-specific ion channel and receptor regulations. This is the key characteristic that distinguishes gintonin from ginsenosides. Although the current discourse on ginseng pharmacology is focused on ginsenosides, gintonin can definitely provide a mode of action for ginseng pharmacology that ginsenosides cannot. This review article introduces a novel concept of ginseng ligand-LPA receptor interaction and proposes to establish a paradigm that shifts the focus from ginsenosides to gintonin as a major ingredient representing ginseng pharmacology.

## Introduction

Ginseng root (*Panax ginseng* CA Meyer) is one of the herbal medicines that have been widely used in Eastern Asian countries for thousands of years. Currently, ginseng is one of the most popular herbs used worldwide (reviewed by Jia and Zhao, 2009). Ginseng history records that it was first considered to be a complementary treatment for “energizing the body” or as a “tonic” and is regarded as a Chinese herbal “Qi” by Asians. Qi in relation to ginseng means energy and life force. Qi was translated by Brekhman into another word in 1966 that describes ginseng is an “adaptogen,” which means, “ginseng extract is believed to increase the body’s ability to resist the damaging effects of stress and promote or restore normal physiological functions” (Brekhman et al., 1966). Currently, the term “adaptogen” is rather vague in terms of modern medicine, and Brekhman et al. (1966) did not provide further information on the active ingredient of ginseng that could play the role of an adaptogen. Instead, the adaptogenic effects of ginseng were translated into the following claims: promotes stamina, increases resistance to disease including cancer, improves physical performance as an ergogenic aid, reduces physical fatigue and mental stress, improves mental awareness, restores and enhances sexual function, and finally, increases life expectancy. However, evidence supporting these claims is still lacking and further studies demonstrating these effects are necessary.

Advanced analytical techniques have revealed that ginseng contains bioactive components such as ginsenosides, acidic polysaccharides, and polyacetylenes as well as other minor components (reviewed by Leung and Wong, 2010). Ginsenosides and their chemical structures were first discovered and identified in ginseng, and are considered as its primary component (Shibata et al., 1965). Ginsenosides are triterpene saponins that are found only in ginseng species. The molecular weights of ginsenosides are in the 1000 Da range, which makes it is easy to isolate them from ginseng (Figure 3). The isolated ginsenosides have a peculiar bitter, sweet, or bittersweet aroma and taste compared to other components. Most ginseng efficacy-related studies have focused on the actions of its saponins (reviewed by Leung and Wong, 2010). However, accumulating evidence show that these identified components of ginseng, especially the ginsenosides, do not represent the complete diversity of ginseng pharmacology. In particular, ginsenosides does not fully exhibit systemic actions *in vitro* and *in vivo* that can be associated with all the underlying molecular mechanisms observed in ginseng pharmacology (Table 1; reviewed by Nah, 2014).

**TABLE 1:** **Summary of comparisons on membrane signal transduction between ginsenoside and gintonin**.

**Ginseng components**	**Ginsenoside**	**Gintonin**	**Reference**
Chemical structure	One of triterpene dammarane glycosides (ginseng saponin)	A complex with lysophosphatidic acid (LPA) and ginseng proteins such as ginseng major latex-like protein 151 and ginseng major storage protein	Hwang et al. (2012), Nah (2014)
Endogenous presence in animals	No	Yes(LPAs present in brain, platelet, serum, and body fluids)	Hwang et al. (2012)
Cell surface membrane binding protein	Interact with ion channels and receptors with non-specific manners	LPA receptors	Hwang et al. (2012), Nah (2014)
Signal transduction pathway systems	No	Pertussis toxin-sensitive and -insensitive G proteins Phospholipase C and IP_3_ receptor activation	Hwang et al. (2012), Nah (2014)
Second messenger production and influence to effector systems	No	[Ca^2+^]_i_ transient induction Ca^2+^-dependent various kinase, ion channels and receptors	Hwang et al. (2012), Nah (2014)
Concentration requirement for *in vitro* cellular responses	High concentration of ginsenoside (M)	Low concentration of gintonin (<nM)	Hwang et al. (2012), Nah (2014)
Capability of intracellular and intercellular communications	No	Yes (through regulations of intracellular ion channels or receptors, glio- or neuro-transmitter release)	Hwang et al. (2015), Kim et al. (2015a)

**FIGURE 1:**
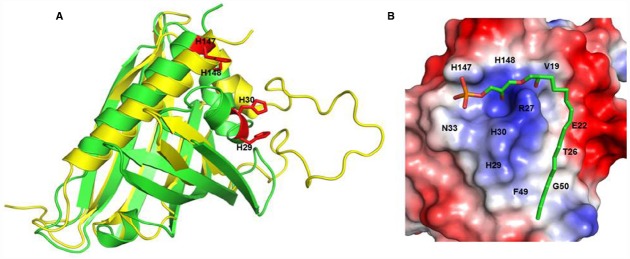
**H146 and H148 are key amino acids for LPA C_18:2_ binding to ginseng major latex-like protein 151.** Recognition of lysophosphatidic acid by ginseng major latex-like protein 151 **(A)** Superposition of ginseng major latex-like protein 151 (GLP; green) and lowest energy major latex protein 28 conformer (yellow). The mutated residues in GLP are represented by red sticks. **(B)** The electrostatic molecular surface of GLP modeled LPA C_18:2_. The positions of the residues that recognize LPA C_18:2_ are labeled. The His147 and His148 residues are important for the interaction between GLP and LPA and for activation of the LPA receptor. Adapted from Choi et al. (2015).

**FIGURE 2:**
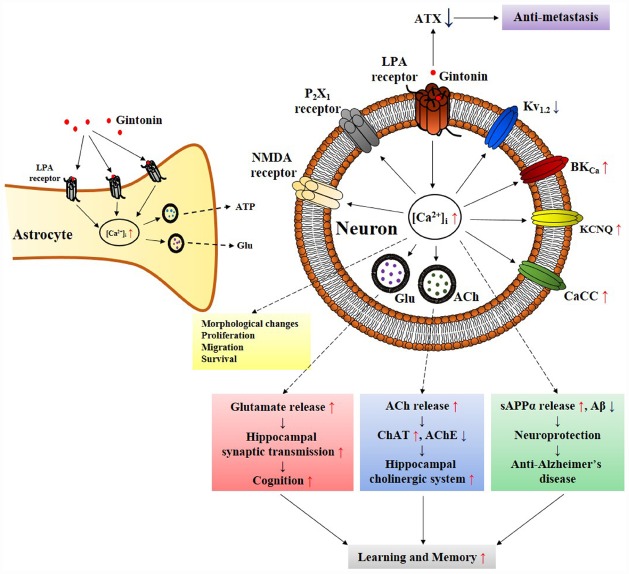
**Schematic diagram that gintonin-mediated ***in vitro*** cellular effects through LPA receptors in neurons and astrocytes is linked to ***in vivo*** pharmacological effects.** The primary action of gintonin produces second messenger Ca^2+^ via LPA receptor activations and regulates Ca^2+^-dependent to various ion channels and receptors regulations, and glio-transmitter and neuro-transmitter release. The ensuing inter-cellular communications via the released neurotransmitters (i.e., acetylcholine or glutamate) can be related to the pharmacological effects that can finally be linked to improvement of learning and memory in nervous system (Kim et al., 2015c). Gintonin also exhibits pharmacological effect against Alzheimer’s disease by attenuating β-amyloid plaque formation and by ameliorating cognitive dysfunction via the activation of non-amyloidogenic pathway and by restoring cholinergic systems that were damaged by β-amyloid in transgenic Alzheimer’s disease animal model (Hwang et al., 2012; Kim et al., 2015b). In astrocytes, gintonin-mediated ATP and glutamate release can be coupled to regulations of neuronal activity (Kim et al., 2015a). In addition, gintonin as exogenous LPA induces various cellular effects such as migration and proliferation of cells as LPAs do through LPA receptor activations. Gintonin also exhibits anti-metastasis activity via inhibition of autotaxin (ATX) activity. ACh, acetylcholine; AChE, acetylcholine esterase; sAPPα, soluble amyloid precursor protein α; ATX, autotaxin; ChAT, choline acetyltransferase; Glu, glutamate.

Other components of ginseng except those noted above are relatively unknown, despite the claims of its diverse efficacy. A key clue suggesting the presence of a novel ingredient in ginseng is the crude ginseng total saponin (cGTS) fraction, which when prepared before further isolation of individual ginsenosides, contains approximately 50% of ginsenosides by weight. The biological properties of the rest of the components of the cGTS fraction besides ginsenosides were previously unknown. Interestingly, the cGTS fraction was shown to mimic G protein-coupled receptor (GPCR) ligands such as acetylcholine by activating the endogenous Ca^2+^-activated Cl^–^ channel in *Xenopus* oocytes (Choi et al., 2001a,b; Lee et al., 2004). Furthermore, the unidentified ingredients in cGTS that induced Ca^2+^-activated Cl^–^ channel activation were not ginsenosides but rather, were similar to an unidentified GPCR ligand (Pyo et al., 2011). Further studies showed that ginseng contains several types of lysophosphatidic acids (LPAs), which are endogenous phospholipid-derived growth factors in animals. In ginseng, the LPAs are isolated as a complex with ginseng proteins that stabilizes and prolongs their activity, and delivers them to their cognate receptors, which is a characteristic that distinguishes them from other plant-derived LPAs (Hwang et al., 2012; Choi et al., 2015; Figure 1). Gintonin, a complex of ginseng LPAs and proteins, activates LPA GPCRs with a high affinity (Hwang et al., 2012). This was the first demonstration that ginseng also contains GPCR ligands. Interestingly, the above traditional claims of ginseng efficacy overlap with the known biological effects of LPAs in many aspects (Table 2). Gintonin via LPA receptor activation provides further systemic evidence of the diverse effects of ginseng that ginsenosides do not (Figure 2; reviewed in Nah, 2012, 2014). The subsequent sections in this review illustrate the differences in the mode of action of ginsenosides and gintonin, and propose a necessary re-establishment of the representative ingredient of ginseng that shifts the claims for the pharmacological properties of ginseng from ginsenosides to gintonin.

**TABLE 2:** **Summary of comparison in *in vitro* and *in vivo* activities of gintonin or LPA**.

	**Gintonin**	**LPA**	**Reference**
*In vitro*			
Receptor	LPA	LPA	Hwang et al. (2012)
G proteins	PTX-sensitive and in-sensitive G proteins	PTX-sensitive and in-sensitive G proteins	Hwang et al. (2012)
Second messenger and effector systems	PLC-IP_3_-Ca^2+^ and Ca^2+^-dependent cellular events	PLC-IP_3_-Ca^2+^ and Ca^2+^-dependent cellular events	Hwang et al. (2012), Nah (2012)
Cellular effects	Proliferation, migration, neurite retraction, and morphological changes	Proliferation, migration, neurite retraction, and morphological changes	Hwang et al. (2012)
Synaptic transmission, gliotransmitter, and neurotransmitter release	Gliotransmitter and neurotransmitter release	Neurotransmitter release	Hwang et al. (2015), Kim et al. (2015a)
*In vivo*			
Nervous systems	Anti-Alzheimer’s disease: attenuation of amyloid plaque formation and restoration of cholinergic system damaged by Aβ	Nervous system development, cognition	Hwang et al. (2012)
Cardiovascular systems	–	Angiogenesis	
Reproductive systems	–	Spermatogenesis and Embryo implantation through LPA3 receptor	
Hair growth	–	Hair growth through LPA6 receptor	
Anti-cancer action	Anti-metastasis though inhibition of autotoxin activity	–	Hwang et al. (2013)

## Functional Ligand of Gintonin but not Ginsenosides is Endogenous to both Animal and Plant Systems

Human beings have isolated and used pharmacologically active, plant-derived ligands for a long time. Some plant-derived ligands mimic the physiological/pharmacological effects of endogenously occurring ligands in animals. However, plant-derived active ligands acting on animal cells usually differ from endogenous ligands in their chemical structures. Interestingly, LPAs are commonly found in both animal and plant systems with the same chemical structure. In animals, Vogt (1957) first found that LPA exists in the intestine and brain lipids. Tokumura et al. (1978) first reported the presence of free LPA in plant soybean lecithin. Plant systems synthesize LPAs and plant-derived LPAs such as gintonin, can serve as LPA GPCR ligands in animal cells, although plant LPAs are merely metabolic intermediates in *de novo* lipid synthesis in plant cell membranes or for glycerophospholipid storage (Millar et al., 2000). Recent studies show that LPA is found in most cell types such as neuronal and glial and non-neuronal cells such as adipocytes and fibroblasts (Tigyi and Parrill, 2003). LPA has also been detected in body fluids such as serum, saliva, and follicular fluid where it performs diverse biological functions (Moolenaar, 1994). LPAs like gintonin exist in both animals as endogenous ligands and plant systems as metabolic intermediates, whereas ginsenosides exist only in ginseng but not animal systems. Although the chemical structure of ginsenosides is similar to that of the steroidal backbone, they are actually triterpenoid saponins that differ from steroids found in animals. Gintonin is an exogenous functional ligand for LPA receptors. This is the first feature distinguishing gintonin from ginsenosides.

## Gintonin Interacts with Proteins on Animal Cell Surface Membranes

Most of animal- and human-derived first messenger endogenous ligands such as hormones or neurotransmitters have binding or interaction protein(s) on cell surface membranes, and each ligand binds to its specific surface protein called a receptor (Kihara et al., 2015). The binding of a ligand to its receptor initiates or translates extracellular information to intracellular sites and transfers extracellular information even at very low concentrations. For example, most hormones and neurotransmitters such as peptides, proteins, catecholamines, and acetylcholine elicit cellular responses at less than nanomolar concentrations (Kihara et al., 2015). Extracellular information may also be transferred to intracellular sites by permeation of ligands into the cell where they bind to intracellular receptors. For example, most steroid hormones act on intracellular receptors (reviewed by Helzer et al., 2015). Animal- or plant-derived bioactive ingredients also exert their effects by acting on their respective receptors located on the cell surface or intracellularly in animal systems. These effects may be agonistic or antagonistic. Human beings have used plant-derived ligands for medicinal purposes since ancient times. Representative plant-derived medicinally active ligands include morphine as an analgesic isolated from the poppy plant and cannabinoids as an anti-glaucoma agent from marijuana, respectively (reviewed by Cichewicz, 2004).

Interestingly, ginseng has a long history of use in traditional herbal medicines but its pharmacology and specific therapeutic applications are not well defined (reviewed in Nah, 2012, 2014). This might be due to a lack of information on the bioactive components of ginseng with the exception of ginseng saponins, which are well characterized (reviewed by Nah, 2012, 2014). Ginsenosides are the first recognized bioactive components of ginseng (reviewed by Nah et al., 2007); however, their current designation as the representative bioactive components of ginseng has several shortcomings. First, ginsenosides have no known specific extracellular or intracellular receptors in animal cells (Nah, 2014), which do not show any spontaneous cellular responses following treatment with ginsenosides. To observe the pharmacological effects of ginsenosides, cells must be pre-stimulated by electrical currents, excitatory ligands, or other treatments or subjected to injuries like hypoxia or ischemia, in the case of organs (Nah, 2014). Second, ginsenosides must be applied at high micromolar concentrations (≈ 30–97 μM in EC_50_ or IC_50_) to elicit any physiological or pharmacological effects compared to other endogenous or exogenous ligands (Choi et al., 2002 Nah et al., 2014). Third, the effects of ginsenosides are miscellaneous, non-selective, and receptor-independent (Figure 4). Ginsenosides lack specific membrane target proteins and interact non-selectively and indiscriminately with various plasma membrane proteins such as ion channels and receptors (Figure 4; reviewed by Nah, 2014). Gintonin as a first messenger isolated from ginseng, binds only to cell surface LPA receptors with a high affinity and elicits cellular responses at less than nanomolar or nanomolar concentration ranges (0.45–18 nM in EC_50_), to primarily activate [Ca^2+^]_i_ transients (Figure 2; Pyo et al., 2011; Hwang et al., 2012; Nah, 2012). This is the second characteristic that distinguishes gintonin from ginsenosides.

## Gintonin has a Specific Signaling Pathway for Activation of Second Messenger

Endogenous or exogenous bioactive ligands activate cell surface receptors and initiate a cascade that amplifies the first messenger action via signal transduction pathways (reviewed by Hofmann and Palczewski, 2015). First, the membrane signaling proteins are activated, and then they mediate subsequent reactions, following membrane receptor activation. Guanosine triphosphate (GTP)-binding proteins are involved in the first step in transferring extracellular information from the first messengers to cytosolic effector systems, which include adenylate cyclase, phospholipase C (PLC), protein kinase C, and others. Finally, the activation or inhibition of effector systems is coupled to final second messenger production or inhibition such as cyclic adenosine monophosphate (cAMP) production or Ca^2+^ release from storage. Endogenous or exogenous bioactive ligands activate receptors that are specifically coupled to cAMP, Ca^2+^, or other second messenger systems depending on the receptor types (reviewed by Clister et al., 2015).

**FIGURE 3:**
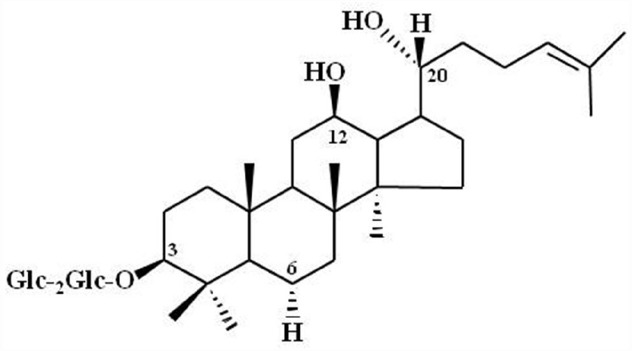
**Chemical Structure of ginsenoside Rg_3_.** Glc, glucopyranoside. Ginsenoside Rg_3_ and other ginsenosides differ in the three side chains and carbohydrates attached to a common steroid-like ring. Subscripts indicate the carbons in the glucose rings that link the two carbohydrates. Adapted from Nah et al. (2007).

**FIGURE 4:**
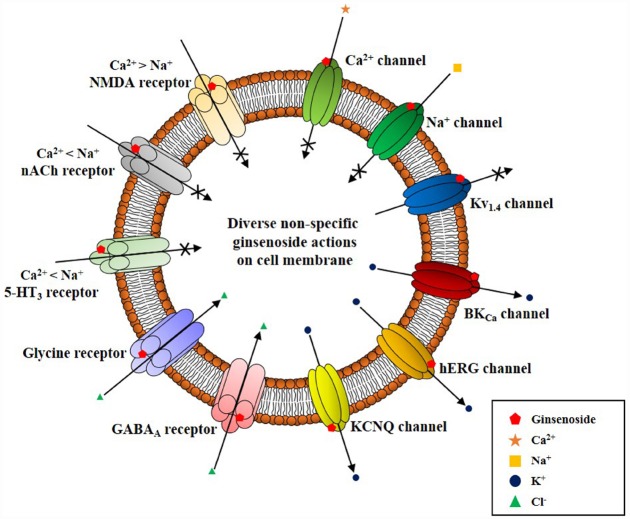
**Schematic diagram on ginsenoside-induced various ion channel and receptor regulations on cell surface membrane.** Ginsenoside (i.e., ginsenoside Rg_3_) actions on cell surface ion channels and receptors show several characteristics. First, ginsenoside shows various non-specific regulations of ion channels and receptors as illustrated here. However, overall actions of ginsenoside decrease the cellular excitability of excitable cells by inhibiting cation influx (i.e., Ca^2+^ and Na^+^ channel activity inhibitions or K^+^ channel activation and ligand-gated ion channel inhibitions such as 5-HT_3_, nACh, and NMDA receptors), and by stimulating anion influx (i.e., GABA_A_ and glycine receptor channel activation). Second, ginsenoside-induced ion channel and receptor regulations achieve via interaction with ion channel pore, channel pore entryway, and share channel blocker or toxin biding sites through site-directed mutagenesis studies (Nah, 2014). Third, ginsenoside itself does not induce ion channel or receptor inhibition or activation at resting state, without preceding stimulations of ion channel or receptors by depolarization or receptor ligand treatment. Thus, the biological or pharmacological effects of ginsenoside could be observed when cells or organs are stimulated beyond normal state rather than receptor mediation like gintonin.

Ginsenosides alone do not elicit any cellular responses related to signal transduction pathways (Nah et al., 1995) and only affect intracellular Ca^2+^ or other cation concentrations when neuronal cells are pre-depolarized or neuronal receptors are prestimulated by excitatory ligands. For example, ginsenosides inhibit NMDA receptor-mediated Ca^2+^ influx only in the presence of NMDA (Lee et al., 2006). Ginsenoside action is limited to the membrane (Table 1; Figures 3 and 4). However, gintonin treatment induces spontaneous responses in cells that express endogenous LPA receptors to elicit [Ca^2+^]_i_ transients. Gintonin modulates membrane signal transduction pathways and effector systems to induce [Ca^2+^]_i_ transients such as pertussis toxin-sensitive and -insensitive Gα_i/o_, Gα_12/13_, Gα_q/11_, and cytosolic PLC and inositol triphosphate (IP_3_) receptors (Table 1;Hwang et al., 2012). Gintonin but not ginsenosides utilizes Ca^2+^ to exert further diverse Ca^2+^-dependent intracellular effects. This is the third feature that distinguishes gintonin from ginsenosides.

## Gintonin Uses Various Effector Systems to Amplify Cellular Effects

Gintonin acts on LPA receptors to activate cells via a transient increase in [Ca^2+^]_i_ levels (Hwang et al., 2012). There are numerous proteins that are dependent on Ca^2+^ for their activation including various kinases, membrane ion channels, and receptors (reviewed by Berridge et al., 1998). Gintonin-mediated induction of [Ca^2+^]_i_ transients is coupled to Ca^2+^-dependent activation of various kinases [such as Ca^2+^/calmodulin-dependent protein kinase II (CaM kinase II), protein kinase C, and tyrosine kinase], ion channels (Ca^2+^-activated Cl^–^, Ca^2+^-activated K^+^, delayed rectifier K^+^, and Kv1.2), receptors (NMDA and P_2_X_1_), hormone secretion (such as dopamine), gliotransmitters [such as adenosine triphosphate (ATP) and glutamate], and neurotransmitter release (such as acetylcholine and glutamate; Shin et al., 2012; Choi et al., 2013b; Lee et al., 2013b; Hwang et al., 2015; Kim et al., 2015a). Ginsenosides do not have a specific signaling pathway for inducing cellular activation and neurotransmitter release. Instead, ginsenosides regulate various ion channels and receptors non-selectively (Figure 4; reviewed by Nah, 2014). Recent studies showed that ginsenosides inhibit ion channels and receptors by directly interacting with amino acids at channel pores similar to channel blockers or toxins (reviewed in Nah, 2014). Ginsenosides simply negatively affect cytosolic ion concentration by cellularly inhibiting Ca^2+^ or Na^+^ influx but by enhancing GABA_A_ and glycine receptor channel Cl^–^ currents (Lee et al., 2008, 2013a; Choi et al., 2009). Ginsenosides have no intracellular mediators that further amplify their effects but regulate ion channel activities by directly interacting with membrane ion channel proteins (reviewed by Nah, 2014). Gintonin but not ginsenosides acts via a second messenger (Ca^2+^) and shows a consistent pattern in its actions that always involves cytosolic Ca^2+^, which mediated its pharmacological effects through various kinases, ion channels, and receptors. This is the fourth characteristic that distinguishes gintonin from ginsenosides.

## Gintonin Mediates Cell–Cell Nervous System Communications

The most important characteristic of endogenous or exogenous ligands is the ability to transfer information from one cell to another to induce subsequent biological effects. Hormones or neurotransmitters play a role in information transfer between cells or from cells to organs (reviewed by Berridge et al., 2003). The elevation of [Ca^2+^]_i_ by GPCR ligand-mediated activation is coupled to neurotransmitter release into the synaptic cleft where it delivers presynaptic information to postsynaptic neurons (reviewed by Berridge et al., 1998). The gintonin-mediated [Ca^2+^]_i_ transient is also coupled to gliotransmitter or neurotransmitter release (Hwang et al., 2015; Kim et al., 2015a). Furthermore, gintonin-mediated release of hormones and neurotransmitters can affect other neighboring or remote cells, resulting in long-term potentiation (LTP) and enhancement of synaptic transmission with subsequent cognitive enhancing effects (Kim et al., 2015c; Park et al., 2015). Therefore, gintonin has specific modulating systems for cell–cell communications via neurotransmitter release in the hippocampus, ultimately leading to biological effects like enhancement of cognitive behavior (Kim et al., 2015c; Park et al., 2015). Ginsenosides themselves do not elicit [Ca^2+^]_i_ transients and are not capable of inducing intracellular and intercellular communications (Nah et al., 2007). This is the fifth feature that distinguishes gintonin from ginsenosides. However, ginsenosides might have several other receptor-independent cellular effects that are not related to cell surface receptor activation. For example, ginsenosides show antioxidant effects and reduce free radical-induced cell damage, which are also observed with most natural products such as fruits and vegetables (reviewed by Liu, 2012). Ginsenosides also inhibit platelet aggregation (reviewed by Mousa, 2010) and enhance non-specific immune responses, which are also observed with most mushrooms with immunomodulatory activities (reviewed by Kang and Min, 2012). The functional comparisons of gintonin and ginsenosides are summarized in Table 1.

## Gintonin but not Ginsenosides has a Clear Mode of *in vitro* and *in vivo* Pharmacological Actions via LPA Receptors

Hecht et al. (1996) first reported LPA as a ligand of the ventricular zone gene-1, which is abundantly expressed in the ventricular zone during mammalian brain neurogenesis. Since then in subsequent studies, six LPA receptor subtypes have been further cloned on the surface of various cells, and most organs also widely express endogenous LPA receptor subtypes (Yung et al., 2015). LPA and its receptors play important roles from the embryonic to adult stage as well as in nervous and non-nervous systems including the brain, cardiovascular, reproductive, and immune systems (Table 2).

Although the LPAs content of gintonin originates from ginseng, gintonin uses the same signaling transduction pathways as animal-derived LPA does in the induction of [Ca^2+^]_i_ transients in neuronal and non-neuronal cells as mentioned above (Hwang et al., 2012). The acute or short-term effects of gintonin but not ginsenosides regulates various Ca^2+^-dependent ion channels and receptors, *in vitro* (Shin et al., 2012; Choi et al., 2013a,b, 2014; Lee et al., 2013b; Hwang et al., 2015). The regulation of ion channels and receptors is coupled to cellular effects. For example, in the nervous system, the gintonin-mediated [Ca^2+^]_i_ transient is coupled to the release neurotransmitters such as acetylcholine, dopamine, and glutamate (Hwang et al., 2015; Kim et al., 2015a; Park et al., 2015). Gintonin-mediated Kv1.2 channel inhibition and NMDA receptor activation are closely associated with LTP induction and enhancement of synaptic transmission in the mouse hippocampus (Park et al., 2015). In addition, gintonin but not ginsenosides stimulates cell proliferation and migration in human umbilical vein endothelial cells and induces neurite retraction via pertussis toxin-sensitive and -insensitive G proteins (Hwang et al., 2012, 2015).

Kim et al. (2015c) observed that hippocampal LTP increased in mice that were previously treated with gintonin for 7 days orally compared to saline-treated mice. In addition, the hippocampi of mice that were previously treated for 7 days with gintonin showed increased expressions of learning and memory-related proteins such as phosphorylated cAMP-response element binding (pCREB) protein and brain-derived neurotrophic factor (BDNF). Finally, in a behavioral study, gintonin administration improved fear memory retention in the contextual fear-conditioning test in mice (Kim et al., 2015c).

Regarding the hippocampal cholinergic system, long-term administration of gintonin to wild-type mice increased the immunoreactivity of hippocampal choline acetyltransferase, which is responsible for acetylcholine synthesis (Kim et al., 2015b). This observation indicates that gintonin treatment not only stimulates acetylcholine release but also induces an increase in the level of the enzyme related to acetylcholine synthesis. In behavioral tests, gintonin treatment also restored scopolamine-induced memory dysfunction in passive avoidance and Morris water maze tests (Kim et al., 2015b). Gintonin showed boosting effects on the brain cholinergic system. Therefore, long-term oral administration of gintonin enhances cognitive functions via activation of cognition related proteins and the cholinergic system.

Astrocytes are considered to have simple structural functions and act as metabolic supporters and protector of neurons in the central nervous system. Recent studies show that astrocytes release gliotransmitters, which modulate neighboring neuronal activities by forming a tripartite synapse with neurons (Araque et al., 2014). LPA receptors are abundantly expressed in astrocytes (Tabuchi et al., 2000; Shano et al., 2008). In addition, astrocytes release LPA in the hippocampus, which then interacts with LPA receptor on neuronal presynaptic sites to induce hippocampal excitation by stimulating glutamate release (Trimbuch et al., 2009). In primary cortical astrocytes, gintonin but not ginsenosides induces a [Ca^2+^]_i_ transient via LPA receptor signaling pathways. Gintonin as well as LPA stimulates the release of gliotransmitters such as ATP and glutamate, and this effect is also [Ca^2+^]_i_-sensitive because [Ca^2+^]_i_-chelators abolish gintonin-mediated gliotransmitter release (Kim et al., 2015a). Therefore, astrocytes produce LPA for release and gintonin as an exogenous LPA source stimulates gliotransmitters via LPA receptors. These results imply that LPA receptors in astrocytes may be positive autoreceptors. The LPA released from astrocytes modulates neuronal activities directly via interaction with LPA receptors on neurons (Trimbuch et al., 2009) or indirectly by releasing gliotransmitter following the activation of astrocytic LPA receptors (Araque et al., 2014). Gintonin as an exogenous LPA source might control neuronal activity in two ways. One way is directly through interactions with neuronal cell surface LPA receptors, and the other is indirect via induction of gliotransmitter release from astrocytes expressing LPA receptors. Therefore, gintonin exert its effects in the nervous system via regulation of neuronal and astrocytic systems through the release of gliotransmitters and neurotransmitters (Figure 2).

## Exogenous Gintonin Exerts Healing Effects in Injured Organs

Lysophosphatidic acid production, release, and receptor expression levels have been observed to change in pathophysiological conditions following cellular or organs injury or trauma. In non-nervous systems, injury to blood vessels activates platelets to release LPA, induces platelet aggregation, and facilitates blood coagulation to stop bleeding (Yoshida et al., 2003; Bolen et al., 2011). Autotaxin (also called lysoPLD, which produces LPA from lysophosphatidylcholine) activity for LPA production increased in the aqueous humor of the eye following corneal damage and ischemia-reperfusion injury of the retina increased LPA release (Liliom et al., 1998; Savitz et al., 2006). Autotaxin activity for LPA production also increased in patients with cancers including breast, melanoma, and ovarian (Leblanc and Peyruchaud, 2015). These observations indicate that the endogenous LPA–LPA receptor system participates in healing processes in blood vessels and the eye. LPA-producing enzymes (autotaxin) are involved in pathophysiological conditions such as cancer progression (Goldshmit et al., 2010). We observed that gintonin stimulated the *in vitro* proliferation and migration of human corneal epithelial cell for wound healing via LPA receptor activation (Hwang et al., 2012). Interestingly, gintonin strongly inhibits autotaxin activity released from melanoma cells, and inhibits cell motility and migration but gintonin had almost no effects on cell proliferation (Hwang et al., 2013). In addition, oral administration of gintonin inhibited metastasis to the lung following administration of cells via the tail vein, and inhibited tumor growth after subcutaneous transplantation of melanoma cells in mice. Gintonin treatment also significantly decreased necrosis, mitosis, pleiomorphisms, and vascularity in tumor tissues (Hwang et al., 2013).

In the nervous system, there have been several reports that LPA concentration and receptor level or autotaxin expression are also altered under pathophysiological conditions in human. Plasma LPA concentration is elevated in patients with ischemic cerebrovascular disease (Li et al., 2008). Human brain neurotrauma caused an increase in LPA receptor expression level (Savaskan et al., 2007; Frugier et al., 2011). In addition, patients with Alzheimer-type dementia showed increased autotaxin expression in their frontal cortices compared to normal brains (Umemura et al., 2006). Treatment with gintonin as an exogenous LPA source exhibited anti-Alzheimer’s disease (AD) activity via LPA receptors. Gintonin-mediated LPA receptor activation is coupled to an increase in the soluble amyloid precursor protein α (sAPPα) release instead of neurotoxic β-amyloid (Aβ) formation in neuroblastoma SH-SY5Y cells but ginsenoside showed no such effects (Hwang et al., 2012). Long-term oral administration of gintonin decreased neuropathies by Aβ plaque formations in the cortices and hippocampi of wild-type mice and restored Aβ-induced memory dysfunctions in a transgenic AD animal model (Hwang et al., 2012).

Acetylcholine is an important neurotransmitter involved in cognitive brain functions such as learning and memory, and the brains of patients with AD show dysfunction of the cholinergic system (i.e., a decrease in brain acetylcholine concentration and choline acetyltransferase activity but increase in acetylcholine esterase, which is an enzyme for acetylcholine hydrolysis). Long-term oral administration of gintonin also attenuated cholinergic dysfunctions in the hippocampus by increasing brain acetylcholine concentrations and choline acetyltransferase activity and decreasing acetylcholine esterase activity (Kim et al., 2015b). Long-term oral administration of gintonin contributed to the restoration of adult brain cholinergic dysfunctions by Aβ itself and Aβ-induced cholinergic dysfunctions in a neurodegenerative AD animal model (Kim et al., 2015b).

Considering the previously described *in vitro* and *in vivo* actions of gintonin, the exogenous application of gintonin may contribute to restoring the condition of damaged cells or injured organs via regulation of LPA receptors and autotaxin activity. The functional comparisons of gintonin and LPA are summarized in Table 2.

## Perspectives and Concluding Remark

The modern interpretations of ginseng pharmacology have advanced tremendously alongside isolation of its components over the last few decades. However, a number of traditional herbal medicine practitioners still believe that the efficacy of ginseng is a mystery. In early ginseng studies in Japan, Russia, and other countries conducted prior to 1960, investigators focused on ginsenosides in an attempt to explain ginseng pharmacology, since they were first identified as components. However, with over five decades of studies on ginsenosides, accumulating evidence shows that the current ginseng pharmacology extends beyond effects that can be attributed to ginsenosides. In other words, attributing the wide variety of pharmacological effects exhibited by ginseng solely to the ginsenosides, is no longer plausible (Figure 3). To advance the elucidation of ginseng pharmacology and development of ginseng-derived medicines, a component with specific targets (i.e., receptor) needs to be identified. Gintonin, which targets LPA receptors unlike ginsenosides, could be a lead candidate for the development of ginseng-derived medicines.

Gintonin, for the first time, can be introduced as a candidate for the novel ligand-receptor interaction concept in the new modern description of ginseng pharmacology. Most therapeutic effects of modern medicines are attributed to ligand-receptor interactions. Currently, studies on gintonin, which is an LPA GPCR ligand, provide diverse evidences of its involvement in the effects of ginseng and, therefore, ultimately explain its pharmacology. In addition, since the activation of LPA receptors by LPA exhibit a variety of biological effects not observed with ginseng, we can further expand ginseng pharmacology by elucidating the gintonin-LPA receptor relationship (Table 2). Furthermore, gintonin could serve as a newly defined up-to-date adaptogenic component of ginseng and a novel candidate to fit the description of “a healing molecule that can restore disruption of physiology functions caused by damage or disease, via LPA receptors or autotaxin regulation.”

Ginseng extract is also considered a complementary and alternative medicine. Currently, more than 50% of medicines used in animals and human beings act via GPCRs, and these receptors along with their ligands are still targets for novel drug development. Many international companies have also concentrated their efforts on the research and development of drugs that act via LPA GPCRs (Llona-Minguez et al., 2015). Gintonin was shown to ameliorate the neurodegenerative AD in an animal model via LPA receptors. Finally, gintonin could be a major lead candidate for development as a ginseng-derived natural medicine and not simply just a complementary and alternative functional food or medicine.

### Conflict of Interest Statement

The authors declare that the research was conducted in the absence of any commercial or financial relationships that could be construed as a potential conflict of interest.
